# Takotsubo Cardiomyopathy in a Young Patient Presenting as Cardiac Arrest and Cardiogenic Shock

**DOI:** 10.7759/cureus.61560

**Published:** 2024-06-03

**Authors:** Adisalem Mulatu Teferi, Hosman Paz, Stanislaw Bankowski, Mona Rahimi, Lynn Zaremski

**Affiliations:** 1 Internal Medicine, St. Barnabas Hospital (SBH) Health System, New York, USA; 2 Emergency Medicine, St. Barnabas Hospital (SBH) Health System, New York, USA; 3 Cardiology, St. Barnabas Hospital (SBH) Health System, New York, USA

**Keywords:** apical hypokinesis, basal hyperkinesis, cardiac arrest, cardiogenic shock, takosubo cardiomyopathy

## Abstract

Takotsubo cardiomyopathy (TC) is a reversible cardiac disorder that rarely results in serious morbidity and mortality. Cardiogenic shock and cardiac arrest can occur in patients with TC. In this case report, we present the case of a 31-year-old woman with no significant comorbidities who suffered cardiac arrest due to TC and subsequent cardiogenic shock that required inotropic and vasopressor support. The patient’s condition progressively improved, and her cardiac function recovered within a few weeks. This case illustrates the importance of recognizing TC as a significant cause of otherwise unexplained cardiac arrest and highlights the need for evidence-based guidelines for treating cardiogenic shock in this setting.

## Introduction

Takotsubo cardiomyopathy (TC), also known as stress-induced cardiomyopathy, is an increasingly recognized medical entity that has a clinical and electrophysiological presentation that may resemble acute coronary syndrome (ACS). It is characterized by significant cardiac dysfunction that typically affects the myocardium in a noncoronary distribution and in the absence of obstructive coronary artery disease [1]. Around 1.2% of patients presenting with troponin-positive ACS have been reported to have TC. Of these patients, 60% have involvement of the apical segment of the left ventricle (LV) [2]. This constitutes typical TC in which there is a predominant dysfunction of the mid and apical segments of the LV. Atypical variants of the disease, on the other hand, typically spare the apex [3].

TC is predominantly a disease of older women, with close to 90% of patients reported to be women with a mean age of 66.4 years [4]. TC is relatively uncommon in non-white racial groups. According to one retrospective study published in the United States, 73.8% of patients admitted with TC were white, whereas Hispanic and Black individuals accounted for only 11.2% and 6.4%, respectively [5].

The pathophysiology of TC remains somewhat unclear. Many triggers have been described, typically involving physiological or physical stresses driven by a catecholamine surge. Additional factors, such as microvascular dysfunction and coronary vasospasm, have also been proposed as possible contributing factors [6,7]. Most patients with TC have predominant systolic dysfunction affecting the apex and/or the mid-left ventricular segments compared to the basal LV. The reason why this happens is not completely understood. One proposed explanation has been the decreasing apical-basal gradient of the beta-2 adrenergic receptors, making the base less susceptible to the adrenergic surge that has been proposed to underlie TC [7]. It has also been described that some patients with TC have dynamic left ventricular outflow tract (LVOT) obstruction, contributing significantly to a reduction in cardiac output. This typically occurs due to the hyperdynamic base causing systolic anterior motion (SAM) of the mitral valve, resulting in significant LVOT obstruction [8].

Our case addresses a rare presentation of TC in a young, relatively healthy, 31-year-old Hispanic female. Additionally, this case was further complicated by the resultant cardiac arrest and cardiogenic shock that ensued in the absence of significant dynamic LVOT obstruction, adding to the atypical presentation of the patient.

## Case presentation

A 31-year-old female patient with a medical history of well-controlled bronchial asthma, migraines, and multiple food allergies was brought in by emergency medical services (EMS) after collapsing. According to the EMS report, the patient, a teacher’s aide, ran toward a fight between students at a school, complained of shortness of breath, and lost consciousness. After she collapsed, bystanders at the school began chest compressions, and the patient was reported to have received shocks from an automated external defibrillator at the scene. It is unclear how many times she received shocks or what the rhythm was at the time of cardiac arrest. The time taken to achieve the return of spontaneous circulation is also unclear. When EMS arrived, defibrillator pads were already attached to the patient’s chest, and she had a pulse, but she was experiencing labored breathing. The patient also had some jerky movements of her extremities and side-to-side ocular movements, which were later described as myoclonus in the ED. The patient never smoked or used drugs or alcohol. She also did not have a family history of premature coronary artery disease or sudden cardiac arrest. Her home medications included albuterol, budesonide-formoterol, ferrous sulfate, medroxyprogesterone acetate (Depo-Provera) injections, and sumatriptan.

On presentation at the ED, the patient had a maximum heart rate of 110 bpm, a blood pressure of 108/55 mmHg, and was saturating at 100% on 15L supplemental oxygen with a non-rebreather mask. She was also found to have a Glasgow Coma Scale of 6-7 with intermittent myoclonic movements. In addition, she was found to have a right lateral brow contusion, clear lungs, and a normal cardiovascular examination without a murmur or gallop rhythm. The patient was intubated for airway protection in the setting of depressed mental status.

The initial blood work was remarkable for leukocytosis, high anion gap metabolic acidosis with elevated lactic acid, and alanine aminotransferase predominant transaminitis, reflecting markedly reduced organ perfusion (Tables 1, 2). The toxicology workup with the urine drug screen and serum alcohol testing was only positive for benzodiazepines, which had been administered in the ED.

**Table 1:** Complete blood count with differentials in the ED

Test	Result	Reference range (units)
White blood cell count	16.7	4.0-10 (10^3^/uL)
Neutrophils	32.1	34.0-71.1%
Lymphocytes	58.6	19.3-51.7%
Monocytes	6.1	4.7-12.5%
Eosinophils	2.6	0.7-5.8%
Hemoglobin	13.6	11.2-15.7 (g/dl)
Platelets	442	150-450 (10^3^/uL)

**Table 2:** Initial comprehensive metabolic panel in the ED

Comprehensive metabolic panel	Result	Reference ranges (units)
Sodium	137	135-145 meq/L
Potassium	4.1	3.5-5.3 meq/L
Chloride	102	96-108 meq/L
Carbon dioxide	17	23-30 meq/L
Glucose	199	70-99 mg/dl
Calcium	9	9.2-11 mg/dl
Urea nitrogen	11	8-23 mg/dl
Creatinine	0.9	0.6-1.2 mg/dl
Total protein	7.5	6.0-8.0 g/dl
Total bilirubin	0.7	0.1-1.2 mg/dl
Aspartate aminotransferase	45	9-33 U/L
Alanine aminotransferase	239	4-36 U/L
Alkaline phosphatase	18	38-126 U/L
Creatinine phosphokinase	103	26-140 IU/L
Lactic acid	8.5	0.0-2.0 mmol/L
Troponin I	0.04	0.0-0.49 ng/ml

**Figure 1:**
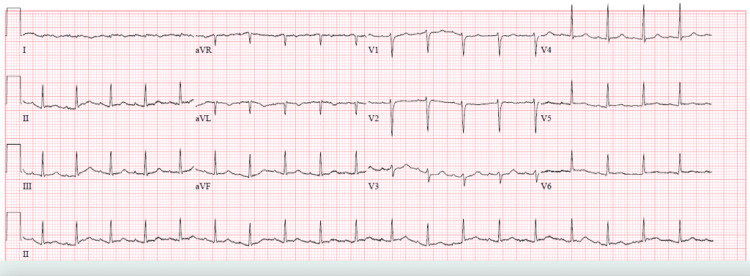
Initial ECG in the ED revealed sinus tachycardia with significantly decreased QRS voltage, especially in the lateral limb leads (leads I and aVL), a pattern that has been described in patients with TC TC, Takotsubo cardiomyopathy

The initial ECG in the ED was remarkable for sinus tachycardia and low-voltage QRS, especially in the lateral limb leads (lead I and aVL), but did not reveal any ischemic changes (Figure 1), and the initial troponin was negative.

After intubation, the patient was persistently hypotensive despite initial fluid resuscitation, so a norepinephrine infusion was started, and she was admitted to the MICU. While in the MICU, she was treated with vasopressors (norepinephrine and vasopressin) and inotropic support with dobutamine to maintain a mean arterial pressure >65 mmHg. She also received dual antiplatelet therapy with aspirin, clopidogrel, and empiric antibiotics. Antibiotics were later discontinued when all infectious workup, including blood culture, was found to be negative.

Additional workups with imaging, including CT of the head, chest, abdomen, and pelvis, were all unremarkable. An EEG performed after the patient was transferred to the MICU revealed a diffuse slowing of cerebral activity, likely related to the cardiac arrest status and sedative medications.

Subsequent measurements of troponin showed an upward trend, which peaked at 4. 61 ng/ml. There were no changes in subsequent ECGs. An ECG performed one day after her MICU admission revealed severe, nearly diffuse left ventricular hypokinesis with preserved function at the base. This was clearly demonstrated with significant apical ballooning during systole, as depicted in Video 1, representing the typical “Takotsubo octopus trap” appearance of the LV and the characteristics of typical TC. The estimated ejection fraction (EF) was 20%. A left heart catheterization performed about 10 days after admission revealed normal coronary arteries, confirming the diagnosis of TC. In the MICU, the patient’s condition progressively improved, and she was extubated after six days of admission and discharged after approximately 12 days of hospital stay with a wearable defibrillator, metoprolol, and furosemide.

**Video 1:** Echocardiography image showing decreased left ventricular function with apical ballooning and associated basal hyperkinesis characteristics of typical TC TC, Takotsubo cardiomyopathy

A week after discharge, the patient was readmitted for easy fatigability and lightheadedness. Evaluation with an ECG and telemetry monitoring did not reveal any abnormalities. A repeated ECG showed an improvement in EF to 60%. The patient’s vital signs were BP 97/62 mmHg and HR 68 beats per minute. Based on these findings, cardiology recommended discontinuing the life vest, metoprolol, and furosemide, and the patient’s symptoms progressively improved off the medications.

## Discussion

TC is an important cause of cardiac dysfunction, with the potential to precipitate cardiac arrest and cardiogenic shock. In addition to the classically described typical TC, also known as the “Takotsubo type” TC, which is characterized by apical ballooning with basal hyperkinesis, there are three other main atypical variants. These include the reverse takotsubo variant, characterized by basal akinesis and apical hypercontraction; the mid-ventricular type, which features mid-LV ballooning; and the localized type, involving akinesis of other segments of the LV. Our patient’s imaging findings are consistent with the typical variant [9].

The diagnosis of TC should be considered in all patients presenting with features of ACS. One of the most widely used diagnostic criteria is the Mayo Clinic diagnostic criteria, which require patients to fulfill all of the following: transient left ventricular dysfunction in a noncoronary distribution; absence of obstructive coronary artery disease on left heart angiography; new ECG abnormalities or elevation of cardiac troponin; and absence of other causes such as pheochromocytoma or myocarditis [10,11].

Cardiac arrest from TC can stem from either cardiac arrhythmias or a sudden impairment of myocardial contractility [12]. Data suggest that at least 9% of afflicted individuals require resuscitative interventions; however, cardiac arrest due to TC is likely underestimated. The incidence of life-threatening arrhythmias in the setting of TC has been found to be between 3.4% and 12.2%. However, these studies do not explicitly delve into the interplay between Takotsubo and cardiac arrest. In some cases, it is difficult to determine if cardiac arrest precipitated TC or vice versa [4].

The largest prospective and retrospective registry, the International Takotsubo (InterTAK) Registry, collected data from 11 different countries and more than 30 centers [13]. Cardiac arrest was rare and occurred in 4.9% of patients. Of these patients who suffered cardiac arrest, ventricular fibrillation (44%) and pulseless electrical activity/asystole (42.9%) were the most common rhythms found at the time of arrest. The remaining patients were found to have ventricular tachycardia (13.1%). TC, presenting as cardiac arrest, is more commonly seen in postmenopausal females with various physical or emotional triggers. The average EF was 33.7%, based on the InterTAK registry. Consistent with previous reports, Jesel et al. found that life-threatening ventricular arrhythmias or cardiac arrest occurred in 23 patients out of 214 cases of TC [14].

Cardiogenic shock is a major contributor to the mortality of patients with TC, especially in the early stages of presentation, and it occurs in approximately 10% of patients [15]. Upon admission to the MICU, our patient was diagnosed with cardiogenic shock and was treated with vasopressors (norepinephrine, epinephrine, and vasopressin) and dobutamine infusions. LVOT obstruction is often the major underlying factor in patients with TC who develop cardiogenic shock. This occurs due to basal hyperkinesis and the SAM of the mitral valve. This can be exacerbated by proximal septal hypertrophy, which is a common finding in older adults [16]. The above considerations make our patient’s presentation with cardiogenic shock in the absence of LVOT quite unusual.

It is crucial to individualize the treatment of cardiogenic shock in the setting of TC, as some treatments indicated for other causes of cardiogenic shock may be detrimental to a subset of patients with TC. When SAM occurs and causes LVOT obstruction due to basal hyperkinesis, cardiogenic shock will worsen when treated with inotropes. These patients may be treated with fluid and/or phenylephrine [17]. The SAM of the mitral valve and LVOT obstruction were not present in our case, and her hemodynamic status improved with dobutamine and a beta-1 active vasopressor.

As mentioned above, we used a norepinephrine infusion in our patient for the management of cardiogenic shock. One important area of uncertainty that will need further investigation is the use of catecholamine vasopressors for patients with TC for treatment of shock, considering the primarily proposed mechanism of catecholamine surge underlying the condition. In the multicenter Registry on Takotsubo Syndrome (RETAKO), for instance, inotropes were used in more patients who died than in those who survived (11% vs. 59%), even if the underlying severity of the disease might have driven the difference in outcome in addition to the difference between the two groups in terms of exposure to catecholamines [18].

Most patients with TC eventually recover, with their left ventricular systolic function returning to normal within one to four weeks. However, the condition is associated with significant in-hospital mortality, which, in some studies, is comparable to that of ACS [4]. The fact that our patient was a young female without prior significant cardiac history likely played an important role in the final outcome and rapid recovery.

Overall, TC, once considered a benign condition, is now increasingly recognized as being associated with complications that can lead to significant mortality and morbidity. Most diagnostic and therapeutic recommendations for the condition are based primarily on retrospective registries and expert opinions rather than controlled clinical trials. According to our literature search, no specific guidelines have been forwarded by major cardiology societies, and further research is required to identify optimal approaches to the clinical care of patients with TC.

## Conclusions

Our case report underscores the importance of considering TC as a potential cause of cardiogenic shock and cardiac arrest, particularly in patients who lack conventional risk factors for coronary artery disease. This can occur even in the absence of significant LVOT obstruction. Moreover, this case report highlights the importance of further clinical research into the optimal treatment of cardiogenic shock and cardiac arrest triggered by TC.
